# Selenium and Dogs: A Systematic Review

**DOI:** 10.3390/ani11020418

**Published:** 2021-02-06

**Authors:** Viola Zentrichová, Alena Pechová, Simona Kovaříková

**Affiliations:** 1 Department of Animal Breeding, Animal Nutrition and Biochemistry, University of Veterinary and Pharmaceutical Sciences, 61242 Brno, Czech Republic; pechovaa@vfu.cz; 2 Department of Animal Protection and Welfare and Veterinary Public Health, University of Veterinary and Pharmaceutical Sciences, 61242 Brno, Czech Republic; kovarikovas@vfu.cz

**Keywords:** nutritional recommendations, blood, serum, Se biomarkers, *Canis familiaris*

## Abstract

**Simple Summary:**

Selenium is a microelement which intake is essential for correct function of the metabolism. In a dog’s body, it is important, for example, for its antioxidant function, its role in thyroid metabolism, synthesis of DNA, or reproduction. It seems that it also plays an important role in prevention and treatment of cancer. While nutritional recommendations for its content in commercial dog food exist, they do not differ between types of food, such as kibble versus canned food. Home-made diets have lower content of selenium than commercial food, but selenium may have greater bioavailability from raw products than processed ones. Moreover, reference values for its levels in dog’s serum, plasma, full blood, and tissues are not very well defined.

**Abstract:**

The intent of this review is to summarize the knowledge about selenium and its function in a dog’s body. For this purpose, systematic literature search was conducted. For mammals, including dogs, a balanced diet and sufficient intake of selenium are important for correct function of metabolism. As for selenium poisoning, there are no naturally occurring cases known. Nowadays, we do not encounter clinical signs of its deficiency either, but it can be subclinical. For now, the most reliable method of assessing selenium status of a dog is measuring serum or plasma levels. Levels in full blood can be measured too, but there are no reference values. The use of glutathione peroxidase as an indirect assay is questionable in canines. Commercial dog food manufactures follow recommendations for minimal and maximal selenium levels and so dogs fed commercial diets should have balanced intake of selenium. For dogs fed home-made diets, complex data are missing. However, subclinical deficiency seems to affect, for example, male fertility or recovery from parasitical diseases. Very interesting is the role of selenium in prevention and treatment of cancer.

## 1. Introduction

Selenium (Se) is a metalloid discovered by Jöns Jacob Berzelius in 1817. It first came into focus in the 1930s, when it was discovered to cause “alkali disease”—a chronic selenium poisoning, appearing in South Dakota and Wyoming, causing horses, hogs, and cattle to shed hooves and lose hair. In chicken, it caused high mortality at hatching and deformities [1]. In 1961, an endemic disease was discovered in China, of which the cause was determined to be selenium intoxication. Symptoms were loss of hair and nails, and in affected villages, it had morbidity up to 50% [2]. On that account, it is not surprising that selenium was looked upon solely as a toxic element for a long time. However, other aspects of selenium slowly started to appear. Schwarz and Foltz [3] proved the essentiality of selenium in experiments on rats—its deficiency caused liver necrosis. Following research showed that offspring of Se deprived rats were almost hairless, grew more slowly, and were unable to reproduce [4]. These discoveries were the start of extensive research about selenium and its importance for health. At present, this research still continues, although it mainly focuses on humans and farm animals. Only a limited amount of information is still available for different species, including dogs, cats, and other carnivores. 

Generally, in a mammal’s body, selenium is important for its antioxidant function, its role in thyroid metabolism, synthesis of DNA, and also reproduction. It is a component of selenoproteins such as glutathione peroxidase (GPx), deiodinases, thioredoxin reductase, and many others [5]. In farm animals, selenium is known to have a narrow window between deficiency and excess, but in dogs, there are no naturally occurring cases of poisoning described. That may be caused by the fact that carnivores preserve higher selenium levels than other species of animals, such as horses, cattle, sheep, or goats [6]. That means there could be many other aspects of selenium metabolism that differ in dogs. The objective of this systematic review was to gather information available on the matter and present a comprehensive report about nutrition and nutrition recommendations, metabolism, biomarkers, and health aspects of selenium in dogs.

## 2. Materials and Methods

For the main body of the article (excluding introduction), a literature search was conducted using the PubMed database. For the combination of words “selenium” and “dog”, up to 14 April 2020, 212 articles were found. Firstly, papers with no abstract (84) and written in a language other than English (2) were excluded. After that, abstracts were read to identify relevant studies, and authors of articles with an unavailable full text were contacted in an attempt to obtain it. Articles covering different problematics other than related to selenium in dogs were excluded. Forty-six scientific texts remained, and when the full texts were read, 3 more works were additionally removed as irrelevant. References of all used articles were checked to find other sources that might not have been available through the database search. That ended up in the total number of 50 scientific texts used for this systematic review.

## 3. Metabolism of Selenium

As most of the research concerning selenium metabolism is focused on humans and farm animals, there is a very limited amount of information regarding Se in canine’s metabolism. Generally, the most significant difference is that dogs as carnivores retain more Se than herbivore and omnivore species as they maintain higher serum levels of selenium [6]. The selenium can enter the body with food in two different forms—organic and inorganic. Organic selenium, mainly in a form of selenomethionine, is received from meat and other “natural” food sources, like viscera or eggs. In contrast, inorganic selenium, usually sodium selenite, is the most frequent form of supplementation used in commercial dog food. Depending on the form, the way of resorption in the small intestine differs. It is believed that while organic selenium has an active transport mechanism (using the pathway of methionine), the inorganic form is absorbed by simple diffusion, and thus more slowly [7]. The bioavailability of sources is frequently discussed, but it just may be that it is not too different in dogs as animals fed high levels of selenium given in form of sodium selenite and organic source had similar fecal absorption [8]. A bigger difference might be made by processing of the diet [9], but the matter will be discussed further in the next chapter.

Selenium is excreted from a dog’s body mainly by the kidneys and can be measured by selenium:creatinine (Se:Crea) ratio. Canines are able to adapt to changes in selenium intake rapidly—in dogs suddenly shifted to low selenium diet (0.11 mg/kg dry matter), Se:Crea ratio decreased by 84% during the first week [10], and dogs supplemented with high doses of both organic and inorganic forms of selenium (8 to 9 mg/kg dry matter) increased their urinary excretion thirty times in 21 days [8]. Some selenium can also be excreted by bile, although the exact amount that can be eliminated this way is unknown.

## 4. Nutrition and Nutritional RECOMMENDATIONS

According to European Union legislation, when selenium is added as a supplement to dog food, the maximal legal limit is 0.5 mg/kg (0.568 mg/kg dry matter—DM). This limit then applies not only to selenium as an additive, but to the total selenium content of the diet—including Se present in ingredients used in manufacturing [11]. Apart from the legislation, there are recommendations that producers of commercial dog foods follow—in Europe, it is the “Nutritional guidelines for complete and complementary pet food for cats and dogs” of The Europe Pet Food Industry [12]. FEDIAF set the minimal limit for adult dogs to 0.35 mg/kg DM for MER (maintenance energy requirement) 95 kcal/kg^0.75^ and 0.30 mg/kg DM for MER 110 kcal/kg^0.75^. The minimal limit for early growth and reproduction as well as for late growth is 0.40 mg/kg DM. This minimal limit was set up following Wedekind et al. [13], who established the minimum requirement based on serum Se in beagle puppies to 0.21 mg/kg DM. Because of the possibility that selenium availability in pet food might be lower than in the study, a safety margin was added, ending in the above-mentioned recommendation of 0.40 mg/kg DM. For adult dogs, the minimum limit is actually set according to the cat’s requirements, as data for dogs does not exist. Taken for the body weight of a dog, the minimal intake of selenium according to FEDIAF is 8.25 µg Se/kg^0.75^.

The Association of American Feed Control Officials (AAFCO) has its own recommendations for commercial dog food. The minimal limit is set to 0.35 mg/kg DM for both mentioned categories—growth and reproduction as well as adult animals. The maximum limit is set to 2 mg/kg DM for regulatory purposes [14]. 

There were a few studies that measured Se content in commercial diets to verify that it is sufficient. Simcock et al. [15] evaluated 37 commercial dog foods, both wet and dry. According to the authors, all of the diets met AAFCO minimal recommendations; there was no significant difference between manufacturers, and mean total selenium was not different between foods produced in Australia, New Zealand, and the USA. The only food that had higher Se content than the rest was a diet with chicken and seafood flavor, but that was probably caused by seafood ingredients. The study also measured cat food, where sea ingrediencies are used more frequently. All foods containing fish and other sea products had higher selenium levels than the rest of diets. Gagné et al. [16] also found selenium levels corresponding with AAFCO recommendations in 45 commercial maintenance diets and 5 therapeutic diets (hepatic and renal). That proves that using commercial diets according to manufacturer’s recommendations ensures sufficient intake of trace elements, including selenium. Another situation arises when the intake of the food is restricted—typically in weight loss plans. There are two theoretical studies, both using hypothetical 20 kg dog, with an ideal weight of 15 kg, that tried to predict nutritional risks of diet restriction during weight loss plans. In the first study, 3 normal commercial diets, and 2 therapeutic weight-loss diets were used for calculation [17]. In the second study, it was 16 normal commercial diets and 15 diets for reduction of bodyweight [18]. The authors of both reports came to the same conclusion regarding selenium and identified it as one of the nutrients affected by the restriction and at the risk of deficiency. Naturally, studies “in vivo” followed the first paper mentioned to verify this statement. Thirty-one naturally occurring obese dogs were fed weight-loss diet according to manufacturer’s instructions. They measured urinary selenium:creatinine ratio in 20 of these dogs, and surprisingly it was higher after weight loss than before it [19]. That might have been caused by degradation of muscle mass, where selenomethionine can figure in the place of methionine. Unfortunately, urinary Se was the only assay of selenium status done in this study. Moreover, there is no information about the diets dogs ate before the start of the study. Thus, we cannot rule out the possibility that when fed the weight loss diet, selenium intake was still higher than from the food used before the study. In another report, 27 dogs fed a commercial diet and undergoing caloric restriction had an average median of selenium intake of just 5 µg/kg^0.75^, which is way below FEDIAF recommendations [20]. Although the dogs stayed healthy the whole time (it lasted 182 to 674 days to reach the ideal body weight), as selenium status was not measured in any way, subclinical deficiency cannot be ruled out. 

The situation is even more questionable when home-prepared foods are used for nutrition instead of commercial diets. Larsen et al. [21] analyzed 39 recipes for homemade diets meant for the management of chronic kidney disease. Thirty-four of these diets had actually lower selenium content than the FEDIAF recommendation. As grave as these results might seem, when considering homemade diets (especially the raw ones) there is a question of selenium availability. It might be better than the availability from commercial food. The first difference is that the main source of Se in kibble commercial diets is usually sodium selenite. That is added as a supplement, while in homemade diets, selenium comes from ingredients and is mainly present in the organic form. The chemical form in which it is present might not play such a large role in dogs—Todd et al. [8] found similar fecal absorption of both sources in animals fed high Se diets. Moreover, urinary excretion and hepatic Se levels were not different between the two sources. The second difference—the process of manufacturing—may, however, be very important. In vitro availability of selenium from different kinds of food was analyzed. Forty-four commercial dog foods, including kibble, pellets, and cans, as well as 8 standard recipes of unprocessed diets were digested in vitro, and selenium was measured in diets and digestates. Raw meat diets had high values—11.73 and 11.96 mg/kg DM, while the median for all diets was 0.45 mg/kg DM. The availability of selenium was 91% in raw diets, 79% in pelleted diets, 58% in canned diets, and 47% in steamed meat. Moreover, in canned diets, availability was lowered by processing, which did not happen in pelleted diets. That indicates that the heating process may reduce availability of selenium. When it comes to the source of selenium, in raw meat and canned diets, it is mainly in the form of selenomethionine, while in pelleted diets, it is mainly as sodium selenite. That may also play a role while processing [9]. When 15 extruded and 8 pelleted Se sufficient dog foods were analyzed for in vitro Se availability, kibble had average availability 71.7% and pellet 78.9%, although the range of both was wide [22]. To prove the hypothesis, that canned diets have a lower Se digestibility than kibble, in vivo study was conducted, using 8 commercial dog foods. Another point of this study was to find out whether selenium availability is affected by crude protein concentration. Four groups, each consisting of six Labrador retrievers, were fed different kinds of diets (each group 4 out of 8 diets used). Canned diets contained selenium only from ingredients, while kibble ones were supplemented with sodium selenite before processing. Blood, urine, and feces samples were collected through the study to assess the impact of diets. Average Se digestibility was 62.3% in kibble and 44.9% in canned diets. However, since the total amount of selenium in cans was almost twice as high as in kibble, total Se intake was still higher from canned diets. Moreover, even though serum Se did not change with the different types of diets, the urinary selenium:creatinine ratio was higher in kibble, meaning more selenium is retained in the body from canned food. That may be caused by the fact that in canned food, selenium is primary organic bound and might have been incorporated in the methionine pool instead of being used for Se metabolism. Moreover, blood glutathione peroxidase was higher in canned diets, but since experimental periods only lasted 29–43 days, it is questionable if that is time long enough for it to react. Crude protein concentration had no effect on the selenium digestibility [23].

## 5. Biomarkers of Selenium Status

The first to try to set reference values for selenium in dogs were Forrer et al. [6]. They measured Se levels in serum of dogs in Switzerland and came with a range of 1.90–4.31 μmol/L for a healthy dog. To our best knowledge, no one else has tried to set reference values since then, but there were studies in which selenium in healthy dogs was measured—often in a group of control dogs. In a control group of 12 Labrador retrievers fed selenium adequate diet [12] for 16 weeks, serum Se was 2.71–3.94 µmol/L [10]. Stowe at el. [24] came with a range of serum Se in Labrador retrievers 2.67–3.10 µmol/L. In their experiment, they used 48 dogs, divided into two groups (full sibling pairs)—one fed a normal diet (slightly selenium inadequate, approximately 90% of recommended minimal level), other getting a 75% restriction of the normal diet (which means about 65% of recommended Se). They found that between 5 and 10 years of age, serum selenium decreases, but the lowest mean value was still 2.67 µmol/L for both groups. 

Fifty healthy adult dogs of various breeds, fed at least partially commercial diets (which gives us at least some presumption of not being gravely selenium deficient) had mean serum Se 3.83 µmol/L [25]. In a control group of 6 healthy beagle dogs, fed a diet that was selenium deficient (containing approximately 70% of the minimal recommended amount of selenium), the mean value of selenium in serum was 3.44 µmol/L [26]. In 14 healthy dogs of various breeds, it was 2.64–4.39 µmol/L [27]. Thirty-eight healthy client-owned dogs, also of various breeds, had levels 2.77–5.14 µmol/L [28]. For the latest two studies, as well as for Forrer et al. [6], there is, unfortunately, no information about the nutrition of dogs, which may cause differences, and it may be the reason for the minimal reference value of 1.90 µmol/L, which seems too low. Animals with such a value might actually be selenium deficient.

Another possibility is to measure selenium levels in plasma. This was done in 49 elderly beagle dogs after being fed selenium adequate diet for four weeks. The range of plasma Se was 2.90–4.31 µmol/L with a mean of 3.49 µmol/L [29]. Eighteen harrier hounds fed a commercial cat diet containing an amount of selenium slightly above the EU legal limit had average plasma Se 3.50 µmol/L [8]. This is again in agreement with values measured in serum. The measured levels of selenium in serum and plasma are summarized in Table 1.

Less information is known about the range of selenium in full blood, as it is not measured as often. That might be unfortunate because a fair amount of selenium is actually present in erythrocytes. Viviano and VanderWielen [30] measured a control group consisting of 14 healthy dogs of various breeds and found a range of 3.17–12.70 µmol/L, with a median of 6.48 µmol/L. However, the range is too wide and the number of dogs too small. Moreover, there is again no information about the diet of dogs. There are another two studies measuring selenium in full blood, but in both of them, control groups were fed diets that were nowhere near FEDIAF minimal recommendations. In the first one, 5 local Chinese dogs, fed a control diet (with inadequate Se content, diet contained only about 30% of recommended Se) for 20 weeks had mean selenium in full blood at just 1.38 µmol/L [31]. The second study had similar results—20 “indigenous” dogs, again from China, were fed “common dry food” (with the measured amount of Se again below even half the recommendation) for 10 days, and had mean values of 1.44 µmol/L in both control and experimental groups [32]. Giving this information into context with other results and nutritional data, it is very likely that dogs in those last two studies were selenium deficient. Another thing pointing towards deficiency is the fact, that in the second study, after supplementation of the experimental group with selenium, mean values raised to 2.68 µmol/L in just 30 days. They might have risen even higher had the study continued longer. The measured levels of selenium in full blood are summarized in Table 2. 

For the measurement itself, various methods were used. Most common was the fluorometric method, as it has the lowest requirements for laboratory and instrumental equipment. Other used methods were high-performance liquid chromatography (HPCL), inductively coupled plasma atomic emission spectrometry (ICP-MS), and atomic absorption spectrometry, either electrothermal (ET-AAS) or with generation of hydride (HG-AAS). As reference materials were used in all of the studies, the results should be comparable.

In other species, glutathione peroxidase (GPx)—a selenoenzyme that protects cells against oxidative damage—is sometimes used as a biomarker of selenium status. Whether that is possible in dogs is still unknown. Either way, if used as a biomarker, it probably does not show current selenium status, but more likely long-term supplementation. Van Zelst et al. [10] found that in Labrador retrievers, when fed a low Se diet (31% of FEDIAF recommendation), glutathione peroxidase in serum and full blood reacts in 6 and 8 weeks, respectively. In the same study, serum selenium lowered during the first week. This is more or less in agreement with other studies. GPx in full blood got higher in 30 days after supplementation with Se [32]. Moreover, it did not change at all in a plasma during a study lasting 21 days, even though some of the animals were supplemented with very high doses of selenium [8].

A few authors measured glutathione peroxidase in their studies. Souza et al. [33] found a significant positive correlation of GPx with Se in both blood and plasma. They were studying 30 dogs positive with Leishmaniosis and 9 healthy controls. On the other hand, Stowe et al. [24] found in their long-term study a positive correlation between GPx in full blood and serum Se only when dogs were between 4 and 6 years old. Significant correlation occurred only in their 9th year. Levels of GPx varied from the start to the end of the 3 months long study in 9 Cairn Terriers [34]. Vajdovich et al. [35] compared serum levels of 14 healthy beagle dogs that were younger than one year, and 14 dogs of the same breed older than nine years. Glutathione peroxidase was twice as high in older dogs than in the young ones. From this data, it is impossible to tell if glutathione peroxide could be used as an indirect assay in dogs, and more studies, mainly ones using bigger numbers of dogs are needed.

Another biologically important selenoprotein is iodothyronine deiodinase. The enzyme is responsible for the conversion of thyroxine (T4) to bioactive triiodothyronine (T3) and the correct function of hormones of the thyroid gland. This suggests using T4 and T3 levels and their ratio as an indirect Se assay. Yu et al. [36] found average serum T3 below the normal range in beagle dogs fed low Se diet after 12 and 24 weeks, but van Zelst et al. [10] did not notice any change in T3:T4 ratio in Labrador retrievers on inadequate Se diet in 8 weeks. It is possible that iodothyronine deiodinase reacts to the depletion of selenium even slower than glutathione peroxidase, and that is why there was no change in the latter study. However, more research is required to understand the relationship between selenium and T3 and T4 levels.

Some authors in their studies also measured selenium in the liver, and although that is probably not very usable in the clinical diagnosis of potentially selenium deficient dogs, results can still be useful. Most of the samples were obtained from necropsies of dogs euthanized for medical reasons in veterinary hospitals. López-Alonso et al. [37] found liver levels in fresh matter (FM) from undetected to 2.55 mg/kg with a median of 0.761 mg/kg in 77 such dogs. Passlack et al. [38] detected selenium levels from 0.098 to 1.909 mg/kg FM with median of 0.426 mg/kg FM in 50 dogs also euthanized for medical reasons. Harro et al. [39] analyzed liver samples from 85 dogs with hepatocellular carcinoma and 85 samples of dogs with no liver pathologies. Control dogs had the median of liver selenium at 2.1 mg/kg FM, while the test group had a median of 2.0 mg/kg FM in non-neoplastic tissue and 1.3 mg/kg FM in neoplastic tissue. According to the authors, this significant difference could have been caused by defective mineral transport or accelerated metabolic rate of hepatocellular carcinoma. In an experimental study (and also the only work that measured liver Se in dry matter), 6 control dogs had mean levels of 1.37 mg/kg DM while dogs supplemented excessively with organic and inorganic selenium had means of 7.93 mg/kg DM and 7.53 mg/kg DM, respectively [8]. That means that the selenium content of the liver is affected by nutrition and that it could be one of the reasons why the previously mentioned authors ended up with such variable results.

## 6. Poisoning

Clinical signs of chronic selenium poisoning from food are anorexia, emaciation, growth retardation, ascites, anemia, coarse and loose hair, and eventual death. When fed a diet fortified with 20 mg of organic Se form (approximately 1.4 mg/kg body weight), neurological disorders can appear, such as walking blindly and stumbling upon objects—similar to “blind staggers” known from farm animals with “alkali disease”. During necropsy, there was a large spleen with hemorrhages, atrophied liver, poorly developed testes, and ovaria in young animals, and sometimes the heart appeared soft and flabby [40]. In this study, intake of 7.2 mg of organic form and 10 mg of sodium selenite was marked as toxic for dogs, but unfortunately, no information on consumed selenium per body weight was available. 

When sodium selenite was applied subcutaneously causing an acute poisoning, the clinical signs were nausea, vomiting, and suffocating before death. While 2 mg/kg subcutaneously injected sodium selenite killed 85% of animals, 1.5 mg/kg killed only occasionally [41]. 

In a more recent toxicity study where Sel-Plex—a selenium yeast product (98% organic selenium), was tested, a NOAEL (no observed adverse effect level—the highest dose at which there was not an observed toxic or adverse effect) lay between 60–200 µg/kg/day of organic selenium for beagle dogs [42]. To our knowledge, there are no records of naturally occurring poisonings of dogs. 

## 7. Deficiency

According to Van Vleet [43], the signs of experimentally inducted combined Se and vitamin E deficiency in 5 to 8 weeks old beagle puppies consist of muscular weakness, subcutaneous edema, anorexia, depression, dyspnea, and later coma. The clinical signs developed after 40 to 60 days. Moreover, there were signs of skeletal degeneration and focal subendocardial necrosis in the ventricular myocardium. It is important to say though, that these were signs of combined deficiency—dogs supplemented with either Se or vitamin E didn’t show clinical signs. Similarly, in a puppy, there was a case of myopathy resembling Se and vitamin E deficiency known from farm animals [44]. When a series of sudden deaths started occurring in a purebred Dachshunds kennel in 1974 and continued sporadically for two more years, the blame was later put on selenium and vitamin E deficiency. It was caused by food that was stored for too long, and after supplementation of Se and vitamin E, the problems stopped. Affected dogs (10 that died in total) all appeared just fine in the evening, but in the morning have been found dead. The only gross changes at the necropsy were hemorrhages on the surface of the pancreas. Histologically, there was hepatic centrilobular congestion, moderate pulmonary congestion and edema, and acute degeneration of the myocardial fibers [45]. The deficiency also had an impact on the immune system, as dog lymphocytes that were incubated with sera from vitamin E and selenium deficient dogs (fed deficient diet from birth to 8 weeks of age) had a depressed response to mitogenic stimulation [46]. Even though these symptoms are sometimes cited as ones caused by selenium deficiency, it is essential to remember that they also always involve inappropriate vitamin E intake.

In beagle puppies, 8 to 10 weeks old, fed a low Se diet (basal diet just 0.04 mg/kg) for 5 weeks, there was only a small change of food intake observed, and no clinical signs of Se deficiency, including body weight gain, were observed [13]. Similarly, adult beagles fed a 0.04 mg/kg Se diet for 24 weeks showed no signs of Se deficiency. The bodyweight and even food intake were not affected. The only sign observed was reduced hair growth in animals fed less than 0.12 mg/kg Se diet—that may be the first sign of deficiency [36]. So, it seems that vitamin E and Se deficiency is synergic in dogs, and Se deficiency alone does not cause such serve clinical signs, at least not on relatively short term basis.

## 8. The use of Selenium in Prevention and Therapy

Different studies were carried out to cast a light on the importance of selenium, its potential therapeutic effects, and the impact of subclinical Se deficiency. One of the researched fields was a reproduction. For example, supplementation with selenium and vitamin E improved sperm quality in dogs with lowered fertility [47] and helped four infertile dogs to mate successfully [48]. Moreover, in normospermic dogs of different breeds, supplementation with selenium, zinc, vitamin E, and folic acid improved multiple sperm parameters [49]. On the other hand, Kirchhoff et al. [34] found no improvement in normospermic Cairn Terriers after supplementation with selenium and vitamin E. It may have been caused by the supplemented doses of both nutrients—in the latest study mentioned, doses per body weight of dogs were way higher, and so instead of having a beneficial effect, could have reached subclinical toxic levels and thus not improving any reproduction parameters.

Most of the attention is probably focused on cancer research, as selenium might be able to play a role in prevention of cancer. In experiments on cell cultures, selenium inhibited the growth of neoplastic cells and induced apoptosis of different tumor cell lines isolated from dogs [50,51,52]. In a mouse model of transplanted canine mammary tumor cells, it also inhibits angiogenesis [53]. Since elderly dogs, unlike most other species, can develop prostate cancer spontaneously similarly to humans, they can be used as a model for preventive programs. Supplementing with selenium causes a lower percentage of extensively damaged prostate cells and increases apoptosis [29]. Moreover, dogs diagnosed with malignant tumors have significantly lower serum Se concentrations than healthy dogs and dogs with different pathologies [27]. In a different study, though, there was no difference between dogs with tumors of the mammary gland, healthy dogs, and dogs with different pathologies [28]. However, this study did not differ between malignant and benign cases of tumors. The most important thing in cancer prevention seems to be the right dose of supplemented selenium. When dogs are over-supplemented selenium then loses its protective role, and there is no additional decrease of prostate cancer risk [54,55]. The theory of human and dog prostate cancer and its “U-shaped dose-response” between selenium and prostatic DNA damage was more profoundly reviewed by Waters and Chiang [56].

Liu et al. [31] studied the effect of Se supplementation of dogs with ethylene glycol-induced calcium oxalate renal calculi. They found out that the supplemented dogs (either with the organic or the inorganic form of Se) have lower urinary calcium levels and milder renal changes. The selenium status also seems to affect progress of parasitical diseases. Dogs supplemented with vitamin E and selenium had better recovery from Sarcoptes infection [57]. In dogs naturally infected with leishmaniosis, plasma Se was lower in infected dogs in comparison to healthy dogs. Moreover, it was lower in symptomatic dogs compared to infected asymptomatic dogs [33]. 

## 9. Summary

The knowledge about dogs and selenium is still incomplete, and there is a lot of room for new research. Starting from nutrition, more information is needed about minimal and maximal limits, and maybe recommendations should take into consideration different types of feed and different availability of selenium from these sources. More research is needed to find out whether homemade diets—both raw and cooked—have a bioavailability of selenium high enough to counterweight its low content of Se. If not, dogs fed these diets can actually be selenium deficient. To assess that, precise biomarkers and reference values of selenium status of dogs need to be set. It seems that the fastest responding and also, for now, most reliable are selenium levels in serum and plasma, and perhaps in full blood. However, for full blood, reference values are nonexistent. The usefulness of glutathione peroxidase as a biomarker is still unknown. When used, it seems to give more long-term information about selenium status, as it reacts more or less with a delay of two months [10]. Other ways of assessing selenium status are, for now, even more unreliable, and their importance will probably not be qualified before exact reference values are set for levels of selenium itself. In clinical practice, selenium seems to be important for male reproduction. It will maybe become a factor routinely considered when dealing with infertile and sub-fertile dogs, but only time will tell. The role of selenium in cancer prevention and treatment is very interesting. That will undoubtedly be researched more profoundly in the future. 

## Figures and Tables

**Table 1:** Levels of selenium in serum and plasma measured by particular authors in their studies.

Author	Sample Type	Number of Dogs	Breed	Age (Years)	Range (µmol/L)	Mean (µmol/L)	Method	Diet (Se Content)	Locality
Forrer et al. [6]	serum	X	X	X	1.9–4.3	X	ET-AAS	X	Switzerland
de Oliveira El-Warrak et al. [26]	serum	6	beagle	mean 3.2	X	3.44	HPCL	Deficient	Canada
Pérez Alenza et al. [28]	serum	38	various breed	5–13	2.7–5.1	3.71	Fluorometric	X	Spain
Pilarczyk et al. [27]	serum	14	various breed	X	2.6–4.4	3.47	Fluorometric	X	Poland
Stowe et al. [24]	serum	48	Labrador retriever	0–10 ^a^	2.7–3.1	X	Fluorometric	Deficient ^b^	USA
van Zelst et al. [10]	serum	12	Labrador retriever	2–6	2.7–3.9	3.26	ICP-MS	Sufficient	UK
Vitale et al. [25]	serum	50	various breed	1–12	X	3.83	ICP-MS	X	USA
Todd et at. [8]	plasma	18	Harrier hounds	2–8	X	3.50	Fluorometric	Sufficient	New Zealand
Waters et al. [29]	plasma	49	beagle	8–10	2.9–4.3	3.49	X	Sufficient	USA

Se—selenium; ^a^—long-term study, dogs measured from early age to 10 years of age; ^b^—two groups of dogs, half getting approximately 90% of FEDIAF recommendation, another half about 65%; X—information is not available; ET-AAS—electrothermal atomic absorption spectrometry; HPCL—high-performance liquid chromatography; ICP-MS – inductively coupled plasma atomic emission spectrometry.

**Table 2:** Levels of selenium in serum and plasma measured by particular authors in their studies.

Author	Sample Type	Number of Dogs	Breed	Age (Years)	Range (µmoL/L)	Mean (µmoL/L)	Method	Diet (Se Content)	Locality
Liu et al. [31]	full blood	5	X	1	X	1.38	HG-AAS	Greatly deficient	China
Ren et al. [32]	full blood	20	X	1	X	1.44	Fluorometric	Greatly deficient	China
Viviano and VanderWielen [30]	full blood	14	various breed	1–12	3.1–12.7	X	ET-AAS	X	USA

Se—selenium; X—information is not available; ET-AAS—electrothermal atomic absorption spectrometry; HG-AAS—hydride generation atomic absorption spectrometry.
